# Application of the advance incision in robotic-assisted laparoscopic rectal anterior resection

**DOI:** 10.3389/fsurg.2023.1141672

**Published:** 2023-03-07

**Authors:** Yuhao Qiu, Ying Li, Zhenzhou Chen, Ninghui Chai, Xianping Liang, Dahong Zhang, Zhengqiang Wei

**Affiliations:** ^1^ Department of Gastrointestinal Surgery, the First Affiliated Hospital of Chongqing Medical University, Chongqing, China; ^2^ Department of Respiratory and Critical Care Medicine, the First Affiliated Hospital of Chongqing Medical University, Chongqing, China

**Keywords:** colorectal cancer, robotic surgery, advance incision, auxiliary incision, laparoscopic rectal anterior resection, operative outcomes

## Abstract

**Background:**

The incidence of rectal cancer is increasing each year. Robotic surgery is being used more frequently in the surgical treatment of rectal cancer; however, several problems associated with robotic surgery persist, such as docking the robot repeatedly to perform auxiliary incisions and difficulty exposing the operative field of obese patients. Herein we introduce a new technology that effectively improves the operability and convenience of robotic rectal surgery.

**Objectives:**

To simplify the surgical procedure, enhance operability, and improve healing of the surgical incision, we developed an advance incision (AI) technique for robotic-assisted laparoscopic rectal anterior resection, and compared its safety and feasibility with those of intraoperative incision.

**Methods:**

Between January 2016 and October 2021, 102 patients with rectal cancer underwent robotic-assisted laparoscopic rectal anterior resection with an AI or intraoperative incision (iOI) incisions. We compared the perioperative, incisional, and oncologic outcomes between groups.

**Results:**

No significant differences in the operating time, blood loss, time to first passage of flatus, time to first passage of stool, duration of hospitalization, and rate of overall postoperative complications were observed between groups. The mean time to perform auxiliary incisions was shorter in the AI group than in the iOI group (14.14 vs. 19.77 min; *p* < 0.05). The average incision length was shorter in the AI group than in the iOI group (6.12 vs. 7.29 cm; *p* < 0.05). Postoperative incision pain (visual analogue scale) was lower in the AI group than in the iOI group (2.5 vs. 2.9 *p* = 0.048). No significant differences in incision infection, incision hematoma, incision healing time, and long-term incision complications, including incision hernia and intestinal obstruction, were observed between groups. The recurrence (AI group vs. iOI group = 4.0% vs. 5.77%) and metastasis rates (AI group vs. iOI group = 6.0% vs. 5.77%) of cancer were similar between groups.

**Conclusion:**

The advance incision is a safe and effective technique for robotic-assisted laparoscopic rectal anterior resection, which simplifies the surgical procedure, enhances operability, and improves healing of the surgical incision.

## 1. Introduction

Currently, the incidence of colorectal cancer and cancer-related mortality ranks third and second, respectively, worldwide (1). For non-metastatic colorectal cancer, surgery is the preferred treatment. Surgical methods have gradually transitioned from traditional laparotomy to laparoscopic surgery and da Vinci robotic surgery. In 2006, Pigazzi et al. (2) described robotic total mesenteric excision (TME) for rectal cancer. Since then, da Vinci robots have been increasingly used for rectal cancer surgery. Because of its more flexible angle, wider field of view, and support of the primary surgeon's hand-eye coordination, the da Vinci robot provides unique advantages with respect to complete mesorectal excision (CME), lymph node dissection, and reduction of intra- and post-operative complications (3–5).

Although the da Vinci robot has clear advantages in robotic-assisted laparoscopic rectal anterior resection, its relatively large size leads to inconvenience for assistants performing procedures such as making auxiliary incisions to remove specimens, placing the tubular stapler anvil, and completing the anastomosis. Therefore, some surgeons choose to dock the robot repeatedly to complete the laparoscopic anastomosis, thus substantially prolonging the operative time (6, 7) and increasing the wear and tear on the robot. In addition, only one assistant assists on the right side of the operating table during robot-assisted surgery. When emergencies occur, such as massive bleeding, rapid conversion to open surgery to stanch bleeding is difficult. Indeed, open conversion increases the risk of surgery because of robot obstruction and a shortage of assistants. In recent years, some surgeons have performed robotic transanal total mesorectal exclusion (R-TaTME or hybrid TaTME) to treat patients with low rectal tumors, obesity, or narrow pelvises (8, 9). However, it still cannot solve the problem of repeated docking and has limitations in the treatment of high rectal cancer.

To increase the appeal of robotic surgery and help promote the use of robotic surgery, we created and introduced the advance incision (AI) for robotic-assisted laparoscopic rectal anterior resection. Before the da Vinci robot is docked, the abdomen is entered in advance by selecting a suitable position, and the robotic surgery is completed with the help of an Alexis wound retractor. In this study, we describe the novel technique and evaluate its feasibility and safety.

## 2. Materials and methods

### 2.1. Law and ethics

This was a retrospective study approved by the Medical Ethics Committee of the First Affiliated Hospital of Chongqing Medical University (No.2022-K398) without the need for participants’ explicit consent. The Institutional Review Board of the First Affiliated Hospital of Chongqing Medical University approved the analysis of patients’ clinical and radiologic data.

### 2.2. Patient selection

This retrospective study was conducted in the Department of Gastrointestinal Surgery at the First Affiliated Hospital of Chongqing Medical University (Chongqing, China). This study followed the Strengthening the Reporting of Observational Studies in Epidemiology (STROBE) statement. A total of 152 patients underwent robotic-assisted laparoscopic rectal anterior resection for colorectal cancer from January 2016 to October 2021.

According to the inclusion and exclusion criteria, 102 patients were included in the study. The patients were divided into AI and intraoperative incision (iOI) groups, with 50 and 52 patients, respectively. All patients underwent surgery performed by the same group of experienced surgeons (>10 years of experience in laparoscopic colorectal surgery and skilled in robotic-assisted colorectal surgery).

The criteria used to select patients were as follows: (1) preoperative pathologic examination showing rectal adenocarcinoma; (2) preoperative magnetic resonance imaging (MRI) showing that circumferential resection margin and extramural vascular invasion are negative; and (3) the operation performed was a curative resection.

The exclusion criteria were as follows: (1) presence of distant metastasis; (2) combined organ resection; (3) robotic-assisted laparoscopic rectal anterior resection with natural orifice specimen extraction surgery; and (4) history of neoadjuvant radiotherapy and chemotherapy.

### 2.3. Operative procedures

After general anesthesia, the patients were positioned in the modified lithotomy position. A robotic-assisted laparoscopic rectal anterior resection was performed with standard techniques, except for the incision-related steps (10). TME and CME principles were followed for all patients (11, 12).

**Figure 1:**
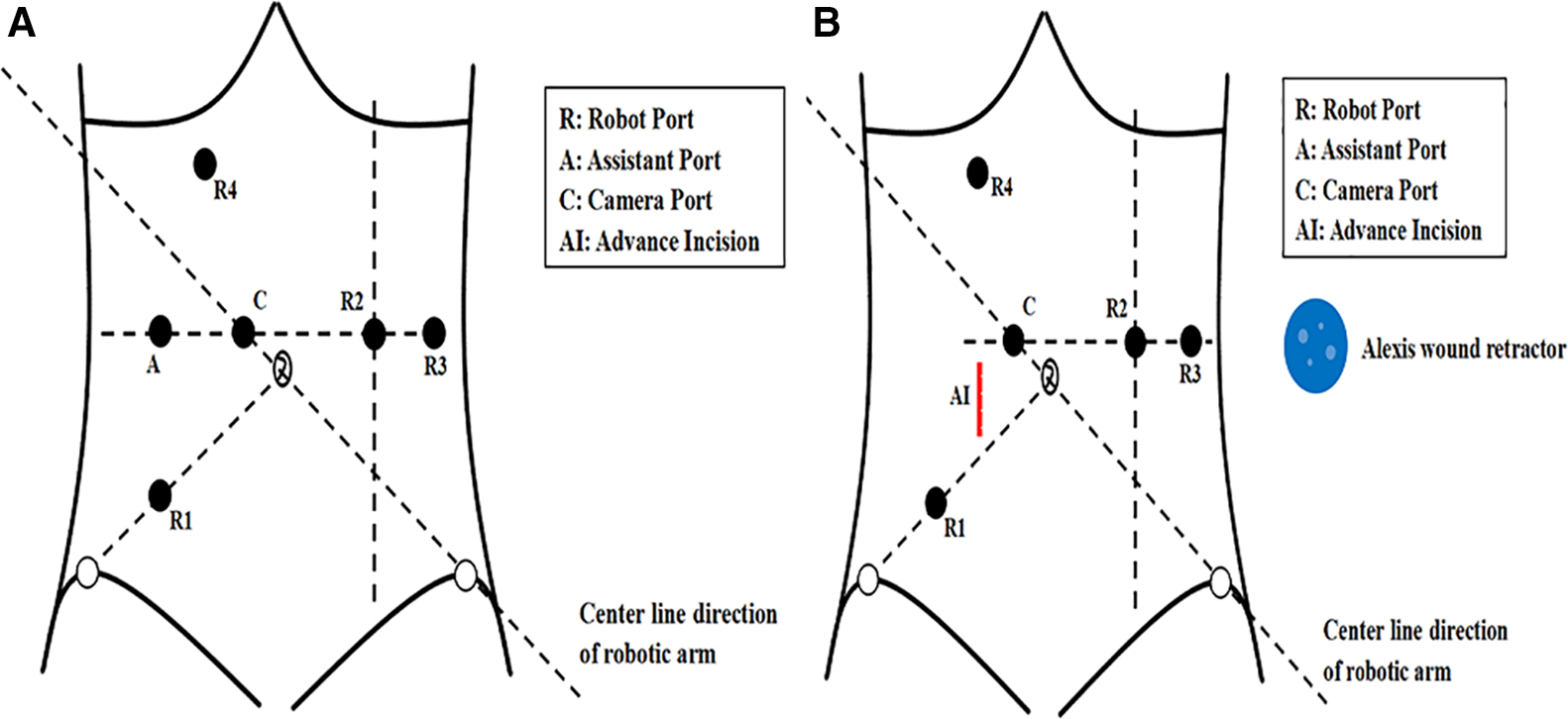
(**A**) The port placement in the intraoperative incision group. (**B**) The advance incision and port placement in the advance incision group.

Placement of the ports in the iOI group was as follows: (1) a 12-mm trocar was placed 3–4 cm superior and to the right of the umbilicus (camera port C); (2) an 8-mm trocar was placed at McBurney's point (robot port R1); (3) an 8-mm trocar was placed at the midline of the left clavicle and horizontal to the camera port (robot port R2); (4) an 8-mm trocar was placed at the left anterior axillary line and horizontal to the camera port (robot port R3); (5) to mobilize the splenic flexure an 8-mm trocar was placed 3–4 cm below the xiphoid process and between the midline and right midclavicular line (robot port R4); and (6) a 12-mm trocar was placed at the vertical line passing through R1 (assistant port A; Figure 1).

**Figure 2:**
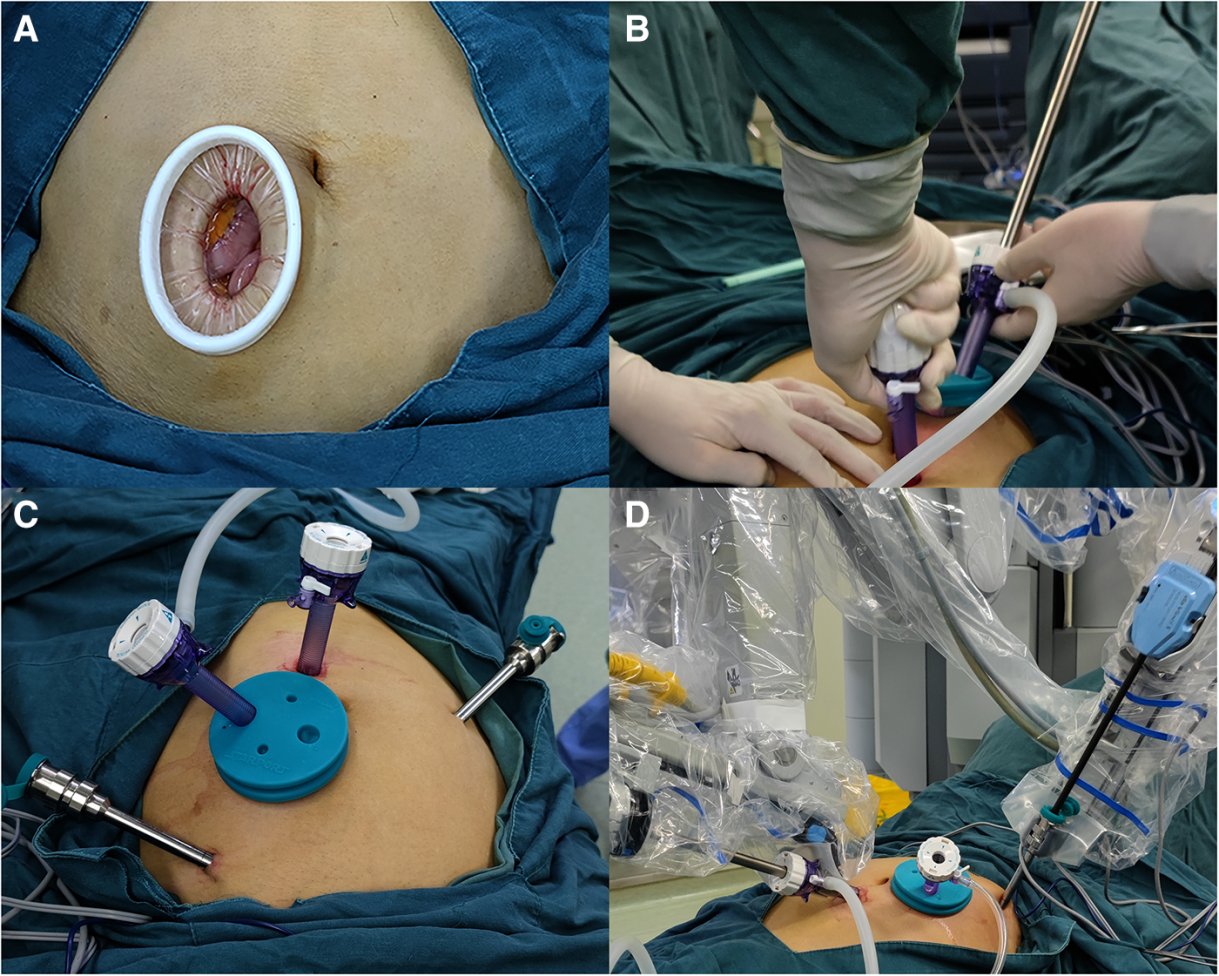
(**A**) The advance incision with an alexis wound retractor. (**B**) Inserting the camera port under direct vision through the advance incision. (**C**) The photograph of the advance incision and the port placement in the advance incision group. (**D**) The photograph of docked robot in the advance incision group.

Before docking the robot, two surgeons made a longitudinal incision through the right rectus abdominis that was 5–7 cm in length and 3–4 cm from the umbilicus in the AI group. The upper edge of the incision was horizontal to the umbilicus. This incision is referred to as the AI (Figure 1B). After the AI was made, a small Alexis wound retractor was placed (Figure 2A). The Alexis wound retractor has an air-tight cover through which a 12-mm trocar was inserted and used as the assistant port. Placement of the camera and robot ports was the same as the iOI group ports (Figures 2B,C). The robot was docked adjacent to the patient's left thigh, and a pneumoperitoneum was established with the pneumoperitoneal pressure maintained at 12 mmHg (Figure 2D).

**Figure 3:**
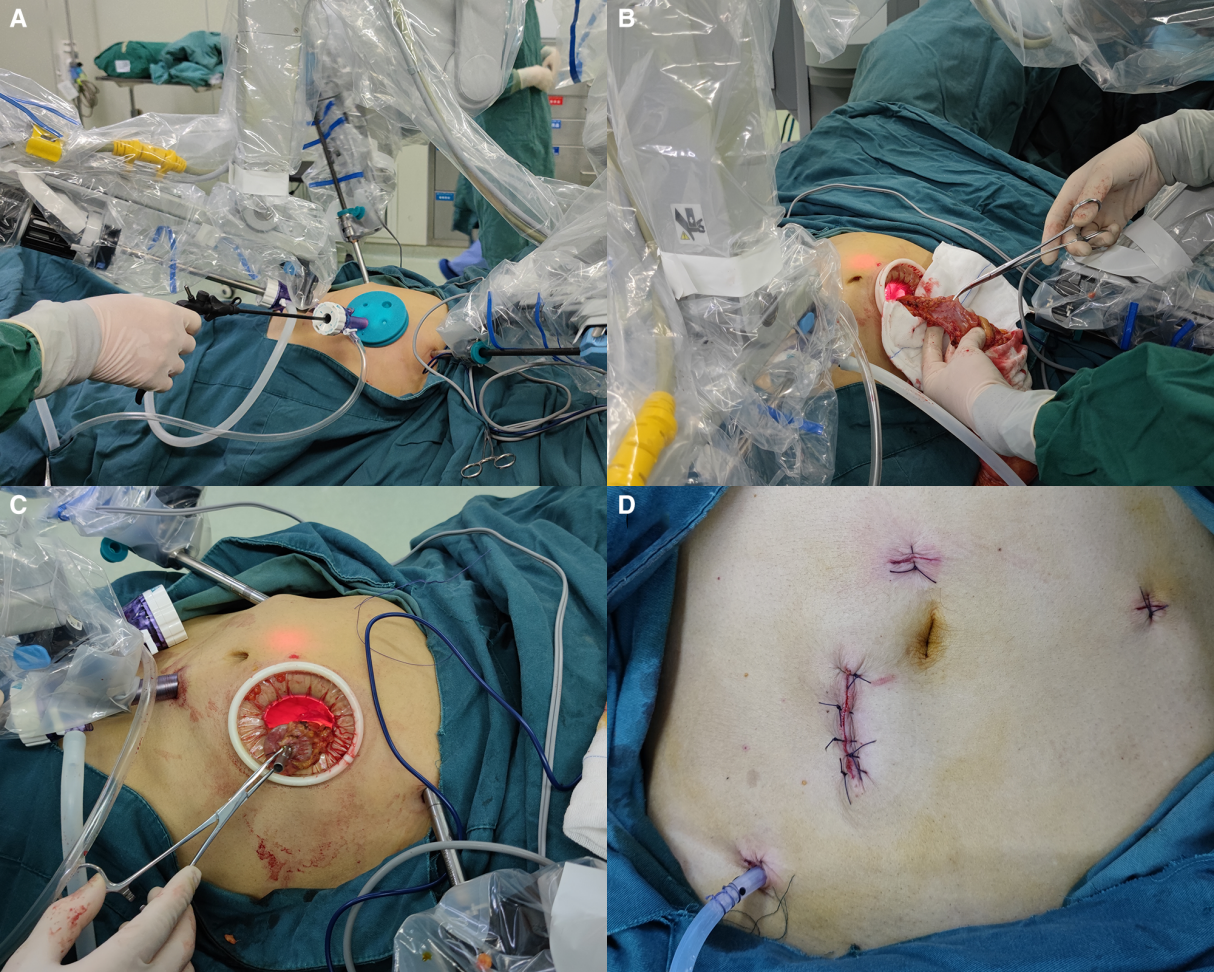
(**A**) The assistant assists in robotic surgery through the advance incision. (**B**) Removal of the specimen through the advance incision. (**C**) Embedding the tubular stapler anvil and replacing the proximal colon into the abdomen through the advance incision. (**D**) The photograph of the advance incision, port incisions, and the surgical drain after suturing.

The surgical assistant stood on the right side of the patient and assisted in the operation through the assistant port (Figure 3A). We used an intermediate approach and performed a colectomy through a medial-to-lateral approach in all patients. Starting from the sacral cape level, the mesentery was stripped upward along the abdominal aorta. Then, Toldt's space was expanded, the inferior mesenteric artery and vein were denuded, and the lymph nodes were excised. For middle or low rectal cancer, the inferior mesenteric artery was ligated distally to the left colic artery to ensure adequate perfusion. The left ureter, gonadal vessels, and autonomic nerves were safeguarded intraoperatively. The colon and rectum were mobilized with an electro-coagulation hook or ultrasonic knife according to the TME and CME principles. A linear stapler (45 or 60 mm) was used to divide the colon at >2 cm from the distal end of the tumor.

When the the previous steps was completed, the pneumoperitoneum was suspended. And we choes not to dock the robot. The assistant port incision was extended according to the tumor size by one assistant, with a length of 3–4 cm, and the Alexis wound retractor was placed in the iOI group. There was no need to make another incision in the AI group. After release of the pneumoperitoneum, the airtight cover of the Alexis wound retractor was removed in the AI group.

The specimen was removed, and the colon was resected approximately 10 cm from the tumor. No ischemia was observed in the proximal colon. Next, a purse string suture was used to embed the anvil of a 29-mm tubular stapler (Figures 3B,C). The proximal colon was replaced into the abdomen, and the airtight cover of the Alexis wound retractor was covered. Finally, a 29-mm tubular stapler was inserted into the anus and anastomosed under direct vision after the pneumoperitoneum was re-established.

The pneumatic leak test was performed by injection of 50 ml of air into the colorectum from the anus to confirm the integrity of the stapled anastomosis. If the pneumatic leak test was positive, additional sutures were placed to strengthen the anastomosis. For patients with a positive pneumatic leak test or an anastomosis <7 cm from the anus, a drain was placed into the rectum through the anus to decrease the rectal pressure. And the drain prevents the faecal load from contacting anastomosis, thereby preventing leakage of faeces into the peritoneal cavity (13). A surgical drain was placed in the pelvic cavity or left paracolic gutter close to the anastomotic site. The peritoneum was then closed with a continuous 2–0 absorbable surgical suture, and the anterior sheath of the rectus abdominis was closed by an interrupted 2–0 absorbable surgical suture. Before being closed, the skin incision was rinsed with 200 ml of dilute iodophor, and the skin was disinfected with iodophor. Finally, the incision was closed with simple interrupted non-absorbable silk sutures (Figure 3D).

### 2.4. Statistical analysis

SPSS statistical software was used for data analysis (SPSS version 26.0; SPSS, Inc., Chicago, IL, United States). Quantitative variables are presented with descriptive statistics, including the median and range. Nominal variables were compared with chi-square test or Fisher's exact test. A *p* value < 0.05 was considered statistically significant.

## 3. Results

### 3.1. Patient characteristics

From January 2016 to October 2021, 152 patients underwent robotic-assisted laparoscopic rectal anterior resection at the First Affiliated Hospital of Chongqing Medical University. Twelve patients underwent combined organ resection, 17 patients underwent natural orifice specimen extraction surgery, 14 patients received neoadjuvant radiotherapy and chemotherapy, and 7 patients were excluded because of incomplete clinical data. Finally, 102 patients were included in the statistical analysis. Among the 102 patients, 50 received an advance incision in the experimental group (AI group), and 52 patients received an intraoperative incision in the control group (iOI group).

The baseline characteristics of the two groups are listed in Table 1. No significant differences were found in sex, age, body mass index (BMI), tumor height above the anal verge, tumor stage, American Society of Anesthesiologists Classification (ASA Class) (14), and prior abdominal surgery between groups.

**Table 1:** Comparison of the baseline characteristics of the study patients.

Characteristic	AI group	iOI group	*p* value
**Number**	50	52	-
**Sex** (male/female)	26/24	27/25	0.994
**Age** (mean ± SD, year)	63.92 ± 11.65	64.29 ± 9.98	0.878
**BMI** (mean ± SD, Kg/m^2^)	23.96 ± 3.86	23.85 ± 2.99	0.869
**Tumor height above anal verge** (mean ± SD, cm)	10.72 ± 6.125	10.73 ± 6.277	0.991
**Tumor stage**			0.612
AJCC I stage	6	7	
AJCC II stage	18	23	
AJCC III stage	26	22	
**ASA class, no. (%)**			0.803^a^
I	2 (4.0%)	4 (7.7%)	
II	23 (46.0%)	21 (40.4%)	
III	22 (44.0%)	25 (48.1%)	
IV	3 (6.0%)	2 (3.8%)	
**Prior abdominal surgery, no. (%)**			0.723
Yes	9 (18.0%)	8 (15.4%)	
No	41 (82.0%)	44 (84.6%)	

SD, standard deviation; AJCC, American Joint Committee on Cancer; No., number; BMI, body mass index; ASA Class, American Society of Anesthesiologists Classification.

^a^ Fisher's exact test.

### 3.2. Surgical procedure

Four patients had positive pneumatic leak tests (AI group, *n* = 2; iOI group, *n* = 2). The anastomosis was reinforced with absorbable sutures, and a surgical drain was placed into the rectum through the anus to decrease the rectal pressure in these patients. All four patients recovered postoperatively, with no anastomosis-related complications. The incision of one patient in the AI group was appropriately prolonged intraoperatively because the tumor was larger than expected; the specimen was removed without difficulty, and no intestinal compression or rupture occurred. The patient did not experience any incision-related complications, such as an incision infection or hematoma. One patient had air leakage of pneumoperitoneum through the AI intraoperatively. The incision was narrowed by sutures, and a large piece of wet gauze was used to wrap the Alexis wound retractor to increase the tightness. The operation was successfully completed.

### 3.3. Comparison of postoperative outcomes

Comparisons of postoperative parameters between patients in the AI and iOI groups are shown in Table 2. No significant differences were observed in the operative time, blood loss, time to first passage of flatus, time to first passage of stool, duration of hospitalization, and rate of overall postoperative complications between groups.

**Table 2:** Comparison of perioperative outcomes between groups.

Parameter	AI group	iOI group	*p* value
**Operative time** (mean ± SD, min)	203.28 ± 52.49	206.94 ± 34.56	0.677
**Blood loss** (mean ± SD, ml)	48.10 ± 31.86	54.92 ± 31.21	0.277
**Time to first passage of flatus** (mean ± SD, day)	2.96 ± 1.38	3.02 ± 2.17	0.870
**Time to first passage of stool** (mean ± SD, day)	4.40 ± 1.78	4.37 ± 2.84	0.942
**Duration of hospitalization** (mean ± SD, day)	8.84 ± 2.91	9.23 ± 4.77	0.620
**Postoperative complications, no.**	8	7	0.717
Anastomotic leakage	1	1	1.000^a^
Blood in stool	1	0	0.490^b^
Bowel obstruction	2	3	1.000^a^
Abdominal infection	2	1	0.972^a^
Chyle fistula	1	2	1.000^a^
Urinary retention	1	0	0.490^b^

SD, standard deviation; No., number.

^a^ Continuity correction.

^b^ Fisher's exact test.

Two patients (one patient in each group) experienced anastomotic leakage. The patient in the iOI group underwent a double-lumen ileostomy because of a severe abdominal infection. The patient in the AI group was cured by non-surgical treatment, including antibiotics, rehydration, and nutritional support. One patient in the AI group had hematochezia at the first day after the operation. The color of the patient's faeces was bright red, and the bleeding volume was approximately 50 ml. The possibility of anastomotic bleeding was considered high. After stanching bleeding with medicines such as Hemocoagulase Bothrops Atrox for Injection, Carbazochrome Sodium Sulfonate for Injection, the patient was cured without anastomotic complications. Except for patients with anastomotic leakage, there were three patients who had positive drainage fluid bacterial cultures (AI group, *n* = 2; iOI group, *n* = 1). The patients had abdominal pain and fever, and the inflammatory indices, including the leukocyte count, and the C-reactive protein and procalcitonin levels, were elevated, thus suggesting an abdominal infection. All three patients were cured after antibiotic treatment. Five patients had bowel obstruction postoperatively (AI group, *n* = 2; iOI group, *n* = 3). Despite the possibility of postoperative intestinal adhesion, the patients recovered after non-surgical treatment, including antibiotics, nutritional support, and gastrointestinal decompression. Three patients had chylous fistula postoperatively (AI group, *n* = 1; iOI group, *n* = 2) and positive chylous tests. The surgical drain was left in place until the drainage fluid was clear and the drainage fluid was <20 ml per day, and the chylous test was negative. Urinary retention occurred in one patient in the AI group postoperatively. Seven days postoperatively, the patient had difficulty urinating after removal of the urinary catheter. B-scan ultrasonography suggested that the residual urine volume was 150 ml. Despite the possibility of a postoperative neurogenic bladder, urinary retention was resolved after bladder training and self-catheterization for 3 months.

### 3.4. Comparison of incisional short-term outcomes

Comparisons of incisional short-term outcomes between patients in the AI and iOI groups are shown in Table 3. The mean time for making an auxiliary incision differed significantly between groups (AI group vs. iOI group = 14.14 vs.19.77 min *p* < 0.05). Moreover, the AI group had a shorter incision length than the iOI group (AI group vs. iOI group = 6.12 vs.7.29 cm *p* < 0.05). In addition, no significant differences were observed in the incidence of incision infection, hematoma, or incision healing time between groups. Six patients developed an incision infection postoperatively (AI group, *n* = 2; iOI group, *n* = 4), and the incision secretion bacterial cultures were positive. We removed the sutures of these patients’ incisions. And we filled the infected incisions with gauze to drain the secretion. With the exception of one patient with an abdominal infection, the other patients were not treated with antibiotics, and all incisions healed uneventfully. Four patients developed incision exudate and were diagnosed with fat liquefaction; their bacterial cultures were negative (AI group, *n* = 2; iOI group, *n* = 2). The patients showed improvement after removing sutures and draining with gauze. Two patients in the iOI group developed incision hematoma postoperatively. We found that the incisions oozed blood and there were subcutaneous hematomas. After removing the sutures, we cleaned the blood clots and sutured to stop bleeding. Then we sterilized and re-sutured the incisions. No incision infections occurred, and the incisions healed well. We routinely scored pain postoperatively [visual analogue scale (VAS) score]. The degree of postoperative incisional pain in the AI group was significantly lower than that in the iOI group (VAS scores: AI group = 2.5; iOI group = 2.88; *p* = 0.048).

**Table 3:** Comparison of incisional outcomes between groups.

Parameter	AI group	iOI group	*p* value
Time of performing auxiliary incision (mean ± SD, min)	14.14 ± 3.04	19.77 ± 2.73	<0.05
Length of the incision (mean ± SD, cm)	6.12 ± 0.68	7.29 ± 0.81	<0.05
Incisional infection, no.	2	4	0.710^a^
Incisional hematoma, no.	0	2	0.496^b^
Fat liquefaction, no.	2	2	1.000^a^
Incisional pain in day 3 (VAS 1-10)	2.50 ± 0.974	2.88 ± 0.963	0.048
Healing time (mean ± SD, day)	7.18 ± 1.79	7.69 ± 2.21	0.202

^a^ Continuity correction.

^b^ Fisher's exact test.

### 3.5. Comparison of survival outcomes between groups

All patients underwent a radical R0 resection, that was confirmed by pathologic evaluation. The mean duration of follow-up was 24.6 months [AI group: 21.2 months (range, 3.0–36.0 months); iOI group: 25.7 months (range, 3.0–39.0 months)]. The overall recurrence and metastasis rates were 4.9% (5/102) and 5.9% (6/102), respectively. The rates of recurrence [AI group vs. iOI group (4.0% vs. 5.8%)] and metastasis [AI group vs. iOI group (6.0% vs. 5.8%)] were similar between groups. Two patients in the AI group (T3N2 and T4N1) and three patients in the iOI group (T4N0, T3N1, and T4N2) developed local recurrence. Three patients in the AI group (T3N1, T4N1, and T4N2) developed hepatic metastasis. One patient in the iOI group (T3N0) had pulmonary metastasis, and two patients (T3N2 and T4N1) had hepatic metastasis postoperatively (Table 4). Tumor staging was performed according to the 8th edition of the American Joint Committee on Cancer Staging Manual (15).

**Table 4:** Local recurrence and distant metastases in the two groups.

TNM stage	Number		Local recurrence		Distant metastasis	
	AI group	iOI group	AI group	iOI group	AI group	iOI group
T1N0	4	4	0	0	0	0
T2N0	2	3	0	0	0	0
T3N0	5	9	0	0	0	1
T4N0	13	14	0	1	0	0
T1N1/2	1	0	0	0	0	0
T2N1/2	3	1	0	0	0	0
T3N1/2	3	5	1	1	1	1
T4N1/2	19	16	1	1	2	1

We also followed long-term incision complications, including incisional hernia and intestinal obstruction. No incisional hernias occurred in the AI group. One patient in the iOI group experienced an incisional hernia 15 months postoperatively. The defect size was approximately 4 cm × 5 cm, and the hernia was reducible. The patient received an abdominal belt without surgical treatment. One patient in the iOI group experienced repeated episodes of intestinal obstruction postoperatively at an interval of approximately 3 months. A CT scan revealed that the obstruction site was located in the pelvic cavity, and indicated an absence of recurrence or metastasis. Because of the possibility of adhesive intestinal obstruction, the patient underwent surgical treatment. No tumor recurrence was observed intraoperatively, however, an adhesive band in the pelvic cavity formed at the obstruction point.

## 4. Discussion

In 2006 Pigazzi et al. (2) reported robotic TME for rectal cancer. In recent years, with continual innovations in robotic surgery technology, robotic surgery has been more widely adopted in patients with rectal cancer; however, because of the relatively large size of robotic equipment, the surgical assistant has limited space to maneuver, thus hindering removal of specimens and placement of the tubular stapler anvil. To simplify the operative process and improve operative efficiency, we developed an advance incision for robotic-assisted laparoscopic rectal anterior resection. Compared with other ways to perform auxiliary incisions in robotic-assisted laparoscopic rectal anterior resection, this technology is safe and feasible, and it effectively improves the prognosis of postoperative wounds, according to our findings. Thus, the advance incision technique has important clinical application value.

We observed no significant differences in the time to first passage of flatus, time to first passage of stool, duration of hospitalization, and rate of overall postoperative complications between groups. These findings might have been because the operations were performed by the same surgeon, and all surgical procedures followed TME standards. The only difference was the sequence of perform incisions, which had little effect on the procedure within the abdominal cavity. The postoperative recovery of patients was not usually affected.

In our study, the overall operative time in the AI group was 206.94 min. Baik et al. (16) have reported an average operative time for da Vinci robot surgery of 217.1 min. Our operative time was shorter, possibly because they undocked the robot to place the tubular stapler anvil (16). Because we have reduced the steps of docking the robot. Therefore, the use of the advance incision effectively shortened the time of robotic rectal surgery comparing with other researches. But the difference between two groups was not significant in total operation time in our study, because the number of times docking the robot was the same. As for the surgical process of the two groups of patients, only the moment of performing the incision was changed, and other operation steps were the same. And performing the advance incision is only a small part of the operation process, which determines that the operation time is more related to the intra-abdominal operation steps. At the same time different patients have different conditions whitch may affect the results of average operation time in each group. Although time of performing the auxiliary incisions was shorter in the AI group, if compared with the overall operation time, its limited difference in the time of performing the auxiliary incisions will be diluted. So there was no statistical difference in the overall time between the two groups, but there was statistical difference in time for performing the auxiliary incisions.

The incidence of postoperative complications was 16.0% and 13.5% in the AI and iOI groups (*p* = 0.717), respectively. The absence of significant differences between groups suggested that the advance incision is safe and feasible. Kang et al. (17) have reported an incidence of postoperative complications in robotic-assisted laparoscopic rectal anterior resections of 19.0%. Alimoglu et al. (18) have reported a postoperative complication rate after robotic rectal surgery of 16.0%. The results of these studies are similar to our results, thus suggesting that the AI is safe and feasible, and does not lead to increased postoperative complications. Herein, the AI group had a significantly shorter time of performing the auxiliary incision, a significantly shorter incision length, and significantly lower postoperative incision pain, possibly because the auxiliary incision was made before docking the robot, thus enabling cooperation among surgeons, superior hemostasis, and better incision healing. The surgeon's intention to shorten the incision due to the fear of air leakage through the advance incision may cause the difference. But the auxiliary incision was performed by one assistant in iOI group. Due to the inconvenience of operating by one person, to better expose the tissue and better stop bleeding, the surgeon may extend the length of the incision involuntarily. Studies have shown that robotic surgery decreases the incision infection rate below that of laparoscopic rectal anterior resection (19, 20). David et al. (21) have reported an incidence of incision infection after robotic rectal surgery of 8.9%. In our study, the overall incision infection rate was 6.0% (4.0% in the AI group and 7.7% in the iOI group). The lower infection rate in the AI group might have been because the AI was closer to the upper abdomen than other auxiliary incisions, and the incision was shorter. Therefore, the advance incision effectively decreases the incision infection rate after robotic surgery and has clinical application value.

The overall postoperative rectal cancer recurrence rate was 4.9%, and the postoperative metastasis rate was 5.9%. Lee et al. (22) have reported a local recurrence rate after robotic rectal cancer surgery of 5.9%, a value similar to our results. Moreover, our study indicated similar recurrence and metastasis rates in the AI and iOI groups, thus suggesting that the advance incision is safe. Although there were differences in the opening time and position of the incision between the two groups, the other operative processes were the same. The two surgical methods complied with the principles of TME and CME for colorectal cancer. Furthermore, we did not observe an increase in implant metastasis in the advance incision, possibly because of the relative distance between the incision and the tumor and the use of an Alexis wound retractor. In addition, opening the incision in advance did not lead to an increase in the incidence of postoperative adhesive ileus, possibly because the occurrence of this condition is related primarily to the operative site and method, but not the time for performing the incision. In conclusion, the advance incision is safe and feasible with respect to long-term complications and prognosis.

It is worth mentioning that we found the following advantages of the advance incision in clinical applications: (1) Before the trocar is inserted into the camera port, in contrast to the advance incision, other surgical methods use blind puncture with the Veress needle, thereby greatly increasing the risk of accidental injury (23). For patients with a history of abdominal surgery, the difficulty and risk of inserting trocars caused by abdominal adhesions are substantially greater. The advance incision can separate the adhesions in the abdominal cavity under direct vision or through the single-port laparoscopic technique (24, 25) before trocar insertion. (2) In the event of an intraoperative emergency, such as uncontrollable massive hemorrhage, the use of an advance incision can notably save the time required for a laparotomy: the advance incision can be extended to achieve rapid laparotomy, and bleeding can even be stopped through the advance incision. For robotic surgery requiring substantial time to docking the robot, the advance incision greatly improves the safety of the operation. (3) The advance incision increases the flexibility of the assistant, allows the assistant to better cooperate with the primary surgeon, and improves the operative efficiency. (4) In obese patients, exposure of the surgical field can be difficult in traditional laparoscopic surgery (26–28). The presence of an advance incision enables intraoperative placement of large gauze to displace organs, such as the small intestine, and help expose the surgical field.

Notably, the following aspects should be considered in the application of the advance incision. (1) The patient's condition should be evaluated in detail preoperatively, the tumor size should be preliminarily evaluated with CT and MRI, and the length of the advance incision should be adjusted appropriately according to the tumor size to avoid excessive extrusion during specimen removal. (2) The advance incision may increase the risk of pneumoperitoneum leakage. If the pneumoperitoneum pressure decreases, the assistant can use a suture to narrow the incision, and wrap the Alexis wound retractor with large wet gauze to strengthen the sealing performance.

This study has several limitations. First, this was a retrospective study with a small sample size. Hence, larger prospective studies are required to confirm the results of this study. Second, the decision to perform an AI or iOI was made by the operating surgeon, thus potentially leading to selection bias; however, all the surgical procedures were completed according to the standard TME surgical procedures; the only difference was the time to perform auxiliary incisions. We believe that the influence of this selection bias was limited and had little influence on the experimental results. Thus, the advance incision is safe and feasible in robotic-assisted laparoscopic rectal anterior resection and has clinical value.

## 5. Conclusion

The advance incision is a safe and effective technique for robotic-assisted laparoscopic rectal anterior resection that simplifies the surgical procedure, enhances operability, and improves the healing of the surgical incision. The application of an advance incision, which may support the promotion of robotic surgery, has important clinical value. Future prospective randomized trials are warranted to validate the findings of our study.

## Data Availability

The raw data supporting the conclusions of this article will be made available by the authors, without undue reservation.
